# The effect of an adenosine A_2A_ agonist on intra-tumoral concentrations of temozolomide in patients with recurrent glioblastoma

**DOI:** 10.1186/s12987-017-0088-8

**Published:** 2018-01-15

**Authors:** Sadhana Jackson, Jon Weingart, Edjah K. Nduom, Thura T. Harfi, Richard T. George, Dorothea McAreavey, Xiaobu Ye, Nicole M. Anders, Cody Peer, William D. Figg, Mark Gilbert, Michelle A. Rudek, Stuart A. Grossman

**Affiliations:** 1 Brain Cancer Program, Johns Hopkins University, David H. Koch Cancer Research Building II, 1550 Orleans Street, Room 1M16, Baltimore, MD 21287 USA; 2 School of Medicine, Department of Neurosurgery, Johns Hopkins University, Baltimore, MD 21287 USA; 3 Surgical Neurology Branch, NINDS/NIH, 10 Center Drive, 3D20, Bethesda, MD 20814 USA; 4 David Heart & Lung Research Institute, The Ohio State University, 374 12th Avenue, Suite 200, Columbus, OH 43210 USA; 5 Heart and Vascular Institute, Johns Hopkins University, 600 N. Wolfe Street, Sheikh Zayed Tower, Baltimore, MD 21287 USA; 6 Critical Care Medicine Department, Nuclear Cardiology Section, NIH Clinical Center, 10 Center Drive, Bethesda, MD 20892 USA; 7 Cancer Chemical and Structural Biology and Analytical Pharmacology Core Laboratory, Johns Hopkins University, Bunting-Blaustein Cancer Research Building I, 1650 Orleans Street, CRB1 Room 1M52, Baltimore, MD 21231 USA; 8 Clinical Pharmacology, NCI/NIH, 10 Center Drive, 5A01, Bethesda, MD 20814 USA; 9 Neuro-Oncology Branch, NCI/NIH, 9030 Old Georgetown Rd, Building 82, Bethesda, MD 20892 USA

**Keywords:** Temozolomide, Adenosine A_2A_ agonist, Regadenoson, Microdialysis, Glioblastoma, High grade glioma, Blood–brain barrier

## Abstract

**Background:**

The blood–brain barrier (BBB) severely limits the entry of systemically administered drugs including chemotherapy to the brain. In rodents, regadenoson activation of adenosine A_2A_ receptors causes transient BBB disruption and increased drug concentrations in normal brain. This study was conducted to evaluate if activation of A_2A_ receptors would increase intra-tumoral temozolomide concentrations in patients with glioblastoma.

**Methods:**

Patients scheduled for a clinically indicated surgery for recurrent glioblastoma were eligible. Microdialysis catheters (MDC) were placed intraoperatively, and the positions were documented radiographically. On post-operative day #1, patients received oral temozolomide (150 mg/m^2^). On day #2, 60 min after oral temozolomide, patients received one intravenous dose of regadenoson (0.4 mg). Blood and MDC samples were collected to determine temozolomide concentrations.

**Results:**

Six patients were enrolled. Five patients had no complications from the MDC placement or regadenoson and had successful collection of blood and dialysate samples. The mean plasma AUC was 16.4 ± 1.4 h µg/ml for temozolomide alone and 16.6 ± 2.87 h µg/ml with addition of regadenoson. The mean dialysate AUC was 2.9 ± 1.2 h µg/ml with temozolomide alone and 3.0 ± 1.7 h µg/ml with regadenoson. The mean brain:plasma AUC ratio was 18.0 ± 7.8 and 19.1 ± 10.7% for temozolomide alone and with regadenoson respectively. Peak concentration and T_max_ in brain were not significantly different.

**Conclusions:**

Although previously shown to be efficacious in rodents to increase varied size agents to cross the BBB, our data suggest that regadenoson does not increase temozolomide concentrations in brain. Further studies exploring alternative doses and schedules are needed; as transiently disrupting the BBB to facilitate drug entry is of critical importance in neuro-oncology.

**Electronic supplementary material:**

The online version of this article (10.1186/s12987-017-0088-8) contains supplementary material, which is available to authorized users.

## Background

The integrity of the blood brain barrier (BBB) is one of the major obstacles to effective chemotherapy for malignant brain tumors. Previous research has focused on how to circumvent the BBB with direct delivery of chemotherapy to the tumor or by mechanically opening the BBB using focused ultrasound or intra-arterial mannitol [1–6]. These direct methods are often associated with comorbidities, hospitalization or added expenses. Very few systemic pharmacologic agents have been evaluated for effectiveness of transient BBB disruption [7–9]. Yet, there is a significant need to identify agents that can transiently disrupt the BBB to improve chemotherapy delivery for patients with such CNS malignancies.

Previous studies have demonstrated the limited permeability of an intact blood–brain barrier [10–12]. However with the presence of tumor cells the BBB becomes heterogeneously disrupted and has been noted as the blood-tumor barrier (BTB) [10]. The BTB and BBB provide a physical barrier with collaborative cells that inhibit entry of toxins, including chemotherapy. Specifically, the BTB amongst malignant gliomas is unique with a high proliferative index of microvasculature and evident alterations in astrocytic endfeet and transcytotic mechanisms; making the BTB more leaky in certain areas of the tumor but peritumoral brain less permeable with a normal BBB [10, 13–15]. These factors collectively play a role in restricting drug entry and have guided extensive research on how best to enhance transport to the CNS.

Adenosine appears to play an important role in the integrity of the BBB [16–21]. The function of adenosine is controlled by four G-protein coupled receptors: A_1_, A_2A_, A_2B_ and A_3_. A_1_ and A_3_ receptors inhibit and A_2A_ and A_2B_ stimulate downstream activation of adenylate cyclase resulting in calcium influx and vasodilation [17, 22]. Inhibitory receptor A_1_ and stimulating receptor A_2A_ exhibit high expression and functionality within the heart and brain; specifically impacting local vasodilation [18, 21, 23]. Regadenoson is an FDA-approved A_2A_ receptor agonist which is routinely used for pharmacologic stress testing in patients with suspected cardiac disease and an inability to perform an exercise stress test. Single-photon emission computed tomography (SPECT) is often performed with a radiotracer to measure myocardial perfusion both at rest and then at the time of stress induced by regadenoson administration. Pre-clinical models have demonstrated the effectiveness of A_1_ and/or A_2A_ receptor agonism to increase BBB permeability to a 70 kD dextran molecule in both mice and rat brains [24]. The large dextran was detected in the brain for up to 180 min following a single injection in both mice and rats. In additional studies that evaluated CNS barrier permeability with regadenoson, there was a 60% increase in temozolomide brain concentrations in non-tumor bearing rats, without changing the systemic pharmacology of temozolomide [19]. These findings prompted clinical studies of regadenoson followed by brain SPECT and CT imaging to evaluate CNS permeability differences, but there was no detectable change in permeability of the BBB in patients [25]. However, no previous study has directly investigated whether regadenoson is capable of increasing temozolomide concentrations in the human brain.

Temozolomide is an FDA approved oral alkylating agent used in newly diagnosed and recurrent high grade gliomas. While temozolomide with radiotherapy has modestly improved overall survival rates in high grade gliomas, previous studies have proven that levels of temozolomide in the brain are only 20% of systemic drug levels [26, 27]. The peak concentration of temozolomide in the brain occurs approximately 1–2 h after ingestion. Once ingested, temozolomide undergoes degradation from its prodrug form to the highly reactive alkylating agent, methyl-triazenyl imidazole carboxamide (MTIC). Previous studies have utilized CSF sampling and intracerebral microdialysis catheters (MDC) to measure temozolomide brain extracellular concentrations in primary or metastatic brain tumors. Use of an indwelling MDC for long term tissue monitoring in the cerebrum is not new, and this technique has been utilized mainly in the traumatic brain injury setting. Prolonged catheter placement allows for continued fluid collections in alert and mobile patients [28–30]. These catheters are often placed in the operating room with verification of placement determined by brain CT. The presence of a gold filament at the catheter tip allows for easy visibility on non-contrast CT brain imaging. The semi-permeable catheter performs similarly to a capillary when perfusion fluid is pumped continuously through it. The presence of the microvial at the end of the catheter allows for regular interval sampling of the dialysate fluid. Then, drug recovery is assessed in each dialysate sample as an indirect measurement of free drug concentration.

Limited clinical studies have been performed in brain tumor patients evaluating drug delivery to the tumor bed using intracerebral microdialysis measurements [26, 31–34]. To date, the only chemotherapeutic agents evaluated have been methotrexate, temozolomide, bafetinib and 5-flucytosine [26, 32, 33, 35]. Portnow and colleagues studied serum and brain extracellular concentrations of temozolomide via MDC collected at 30 min time intervals post oral drug administration over 24 h. This study evaluated temozolomide drug delivery to the peritumoral non-contrast enhancing area in both primary and metastatic patients (n = 10). Collectively, they found that oral administration of temozolomide yielded an average brain:plasma AUC ratio of 17.8 ± 13.3%, with a peak drug concentration of approximately 2–3 h after administration and undetectable concentrations by 18 h [26]. We designed this study to determine if FDA-approved doses of regadenoson would increase the temozolomide concentration in human brains with malignant glioma as measured by serial brain interstitial fluid assessments.

In this pilot feasibility study, we utilized MDC to determine the neuropharmacokinetics of temozolomide co-administered with regadenoson to assess temozolomide drug entry. We hypothesized that regadenoson would transiently impact the permeability of temozolomide as it did in rodents resulting in increased brain interstitium (BI) and brain:plasma AUC ratio. The primary aim of this trial was to measure brain interstitial temozolomide concentrations pre and post regadenoson using MDC in patients with recurrent high grade glioma. The secondary endpoint was to evaluate tolerability of temozolomide with a single dose of regadenoson in the post-operative setting.

## Methods

### Study subjects

This study was approved by Institutional Review Boards at Johns Hopkins and the National Institutes of Health, and all patients provided informed consent. Eligible patients were ≥ 18 years old with a diagnosis of recurrent high grade glioma suspected by MRI findings. All patients had a clinically indicated need for surgical intervention. Patients were required to have: Karnofsky performance status (KPS) of ≥ 60%; normal liver and kidney function; absolute neutrophil count ≥ 1500 cells/mm^3^; and a platelet count ≥ 100,000 cells/mm^3^. Patients were excluded if they were currently receiving chemotherapy or radiation therapy, allergic to temozolomide, pregnant or breast-feeding, had a serious medical or psychiatric illness or social situation that could interfere with catheter placement/monitoring. Patients with a prior use of VEGF or VEGFR-targeted therapy, use of investigational agents within the past 4 weeks, NCI CTC grade 3 or greater baseline neurologic symptoms, history of cardiac, bronchospastic lung disease, or a contraindication to adenosine were all excluded from study participation. Additionally, patients were asked to refrain for caffeine use at least 24 h prior to regadenoson administration, secondary to its ability to blunt the effect of regadenoson.

### Study design

Once intra-operative pathology was confirmed, one to two MDialysis 70 Microdialysis Brain Catheters (membrane length 10 mm; shaft length 60 mm; ref. no. P00049) were placed into contrast-enhancing and/or non-contrast peritumoral tissue (within 5 mm from the resection cavity). Post-operative non-contrast CT imaging confirmed catheter placement with identification of an enhancing gold tip. After transfer to the intensive critical care unit, the inlet tubing of the catheter was connected to a portable syringe pump (MDialysis 107 Microdialysis Pump, ref no. P000127), containing artificial CSF (Perfusion Fluid CNS, ref no. P000151) at a rate of 1 µl/min. A microvial was connected at the end of the outlet tubing to continuously collect dialysate samples. To account for the correction factor, fractional recovery of temozolomide by ICMD was calculated based on previous in vitro sampling using CMA 70 Microdialysis catheter [26]. All microdialysis supplies were purchased from MDialysis, (Stockholm, Sweden).

Once the patient was clinically stable, at least 24 h after the completion of surgery on post-operative day 1, and tolerating oral intake, they were administered temozolomide 150 mg/m^2^ orally once. On post-operative day 2, each patient was again given temozolomide 150 mg/m^2^ orally, and approximately 60 min later, each patient was administered intravenous regadenoson 0.4 mg once over 10 s. Previous studies have demonstrated temozolomide peak brain concentrations occur 90–120 min after administration [26, 27]. Additionally, preclinical studies with regadenoson have demonstrated the peak effect on barrier permeability occurs 30–60 min after administration [19, 24, 36]. Thus, we opted to administer regadenoson 60 min after temozolomide to ensure peak concentration of temozolomide in the brain and optimal mechanism of regadenoson action on brain vasculature simultaneously. Regadenoson was given with continuous ECG monitoring for a total of 10 min post injection, in the presence of a staff cardiologist (standard cardiac dosing regimen). Pre-temozolomide blood samples were collected 15 min prior to drug administration and then 1, 2, 3, 4, 8, and 18 h after the dose of temozolomide (5 ml per collection). The samples of blood were collected in heparinized syringes, promptly mixed by inversion, and then placed on wet ice until centrifugation at 1300×*g* for 10 min at 4 °C (within 1 h). The samples were processed to plasma within 30 min from centrifugation, and the pH of each sample was adjusted to < 4 with the use of 8.5% phosphoric acid. Plasma was then stored frozen at − 70 °C or below until subsequent batch analysis via liquid chromatography-tandem mass spectrometry (LC–MS/MS).

Dialysate samples were continuously collected with microvial changes every 3 h after portable syringe pump connection on post-operative day 0. At least 24 h after surgery, the microvial was changed pre-temozolomide ingestion and then 1, 2, 3, 4, 6, 8, 10, 12, 14, 16, and 18 h after temozolomide intake on post-operative day 1 and 2. For temozolomide stabilization each microvial was prefilled with 6–12 µl of acetic acid. Microvials containing dialysate samples were stored on dry ice until all microdialysis samples were collected from the patient. Thereafter, the dialysate samples were stored at or below − 70 °C until LC–MS/MS analysis.

### Analytical method to evaluate temozolomide concentrations

Temozolomide concentrations were quantified in acidified sodium heparin plasma and acidified brain interstitial dialysate. For plasma, 15 µl of 8.5% phosphoric acid was added per 0.5 ml of plasma. For microdialysis fluid, 1 µl of glacial acetic acid was added for every 10 µl of the dialysate. Perfusion fluid CNS (artificial CSF) was used as a surrogate matrix for dialysate standards and QCs. Temozolomide was extracted from samples (50 µl of acidified plasma or 20 µl of acidified microdialysis fluid) by adding 600 µl of ethyl acetate containing IS (20 ng/ml of caffeine-^13^C_3_) (Sigma–Aldrich, St. Louis, MO). Samples were vortex-mixed and centrifuged at 2000 rpm for 10 min. The top layer was transferred to a clean glass tube and dried under a nitrogen air stream. Samples were reconstituted with 200 µl of 0.5% formic acid in water and stored in the autosampler at 5 °C for LC–MS/MS analysis.

Liquid chromatography-tandem mass spectrometry analysis was performed on an AB Sciex 5500 QTrap mass spectrometer (Sciex, Foster City, CA) coupled with an Acquity UPLC system (Waters, Milford MA). The LC separation was achieved using a Zorbax XDB C_18_ column (4.6 × 50 mm, 5 µm) (Agilent, Santa Clara, CA) at room temperature. The mobile phase solvent A was water containing 0.1% formic acid and mobile phase solvent B was methanol containing 0.1% formic acid. The mobile phase was delivered at a flow rate of 0.3 ml/min. The initial mobile phase composition was 60% solvent A and 40% solvent B. From 0.5 to 4.0 min, solvent B was increased to 100% and conditions held until 5.0 min. At 5.1 min, the mobile phase composition was then returned to 40% solvent B until 6.0 min. The total runtime was 6 min.

The column eluent was monitored using a Sciex 5500 QTrap mass spectrometer using electrospray ionization operating in positive mode. The mass spectrometer was programmed to monitor the following multiple reaction monitoring (MRM) *m/z* transitions: 195.15 → 138.10 and 198.00 → 140.00 for temozolomide and IS, respectively. Calibration curves for temozolomide were computed using the area ratio peak of the analysis to the internal standard by using a quadratic regression with 1/x^2^ weighting for plasma and 1/x weighting for artificial CSF, both with a calibration range of 0.005–1.0 µg/ml, and dilutions up to 1:10 (v/v) were accurately quantitated.

### Pharmacokinetic analysis

Using non-compartmental methods, the pharmacokinetic parameters within plasma and dialysate temozolomide were determined by concentrations vs. time. While the maximum concentration (C_max_) and time maximum concentration (T_max_) were determined directly from the measured data points, half-lives (t_1/2_) were calculated from the elimination rate constant derived from the terminal slope. The AUC_0–18 h_ for each day was estimated by standard non-compartmental analysis performed by Phoenix^®^ WinNonlin version 6.3 (Pharsight Corporation, Mountain View, CA, USA). The pharmacokinetics variables were tabulated, using descriptive statistics calculated pre and post regadenoson. The differences of the AUCs were summarized by mean and standard deviation. Means and standard deviations were presented for peak drug concentration (C_max_), time of peak drug concentration (T_max_), drug half-life (t_1/2_) and area under the curve from time 0–18 h after temozolomide administration (AUC_0–18 h_).

## Results

### Feasibility/safety and tolerability

**Fig. 1:**
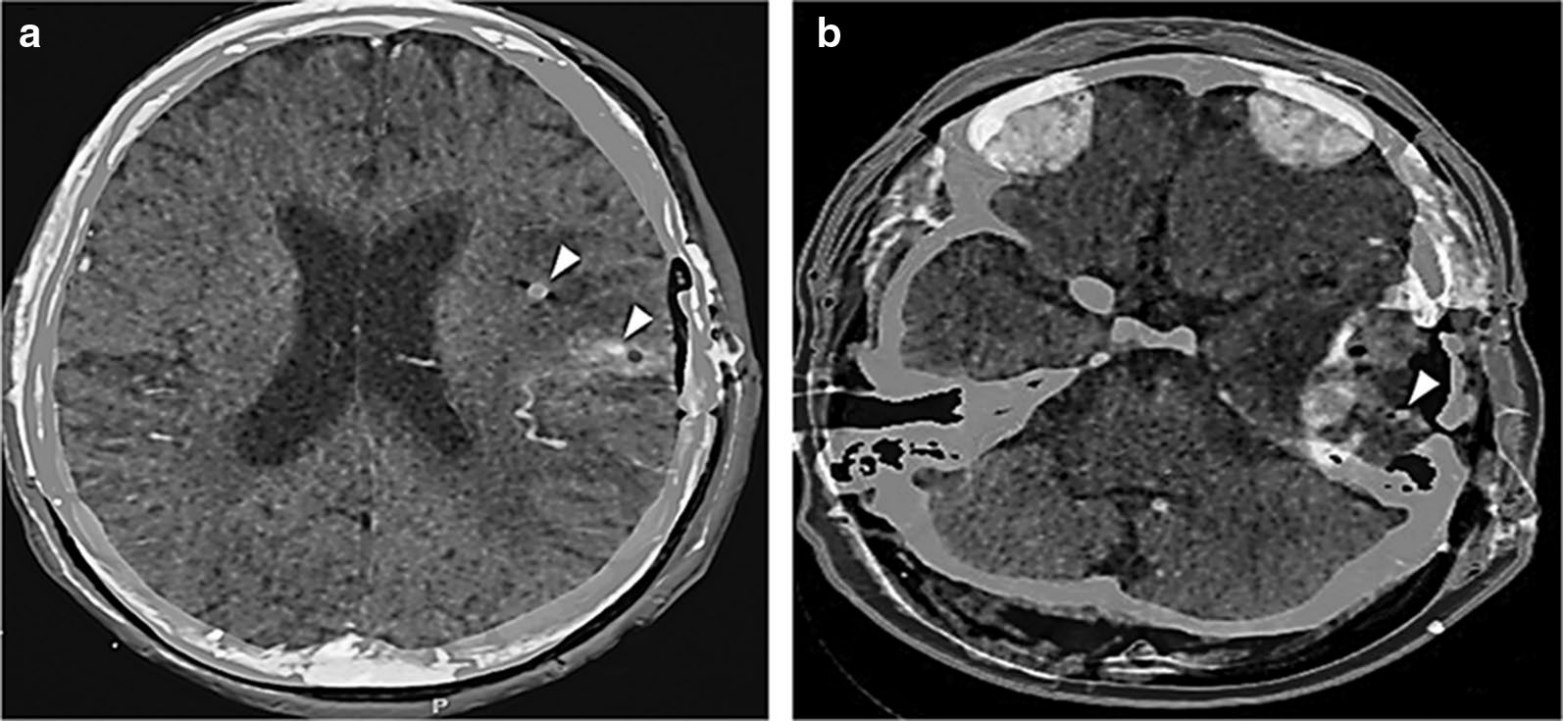
Catheter placement imaging. Brain CT and MRI superimposed delineating catheter placement and tumor margins. Patient 1 had one catheter tip placed in non-contrast enhancing area and the second in contrast enhancing tissue (**a**). Patient 2 had one catheter placed in non-contrast enhancing tissue (**b**). Superimposed images denote contrast enhancement in green. The white triangle indicates placement of catheter tips

Six patients were enrolled on the study from May 2015 to April 2017. Five of the patients were deemed as evaluable. One patient was deemed unevaluable due to microdialysis catheter displacement, which occurred approximately 24 h after insertion. This displacement was attributed to manipulation of the patient’s surgical head dressing post-operatively. This patient was removed from study before any study medications were administered. Table 1 summarizes patient demographics. All patients underwent surgical debulking for their recurrent high grade glioma. Each catheter was placed in peritumoral tissue that was deemed to be non-contrast enhancing tissue by pre-operative MRI. In two patients, catheters were also placed in contrast enhancing areas approximately 5 mm from the surgical bed due to a subtotal resection. Catheter tip locations were accurately determined by superimposed CT and MRI imaging (Fig. 1). All evaluable patients tolerated microdialysis catheter insertion without associated bleeding, infection, pain or other incidents attributable to foreign material placement.

**Table 1:** Clinical demographics

Patient	Age	Gender	Diagnosis	Catheter(s) tip placement area
1	49	M	Glioblastoma	Non-contrast and contrast enhancing
2	68	M	Glioblastoma	Non-contrast enhancing
3	32	M	Glioblastoma	Non-contrast and contrast enhancing
4	60	M	Glioblastoma	Non-contrast enhancing
5	51	M	Glioblastoma	Non-contrast enhancing
6	70	F	Glioblastoma	Non-contrast enhancing

All patients diagnosed with recurrent glioblastoma and catheter tips were placed in non-contrast enhancing tumor. In patients 1 and 3, tips were also placed in contrast enhancing tissue

Post-operatively, each patient was transferred and cared for in the intensive care unit during the entire duration of the microdialysis sampling period. Dialysate samples were obtained from 5 patients, with sampling obtained for approximately 72 h from catheter insertion. Drug administration and monitoring while in the ICU included regadenoson administration by the trial-associated cardiologist. Each patient received temozolomide 150 mg/m^2^ daily for 2 days and on day 2 was administered regadenoson by the cardiologist; who noted expected regadenoson side effects of transient tachycardia, elevated blood pressure and flushing. Only two patients experienced grade 1 headaches, (patient 2 and 4) which resolved within 30 min from regadenoson administration. No subjects required aminophylline as a reversal agent to regadenoson. No unanticipated adverse events were noted from temozolomide, regadenoson drug administrations, or microdialysis catheter sample collections.

Dexamethasone was administered to each patient as part of their standard post-operative care; in an effort to minimize post-operative vasogenic brain edema. For each patient on study, dexamethasone was given via intravenous administration every 8 h. Specifically, on the days of study, all patients except patient 4 received dexamethasone approximately 2 h after temozolomide administration (1 h after regadenoson administration). For patient 4, dexamethasone was given at the same time as temozolomide (1 h prior to regadenoson administration).

### Temozolomide neuropharmacokinetic analysis

**Fig. 2:**
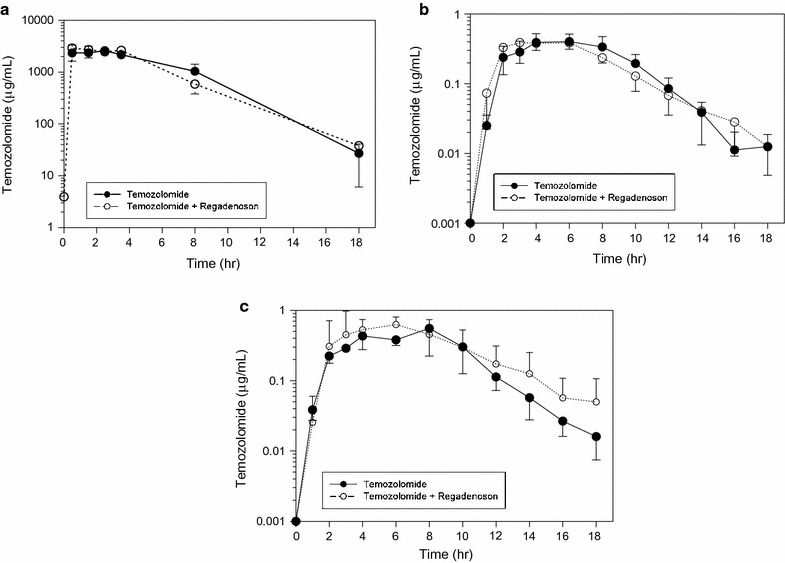
Mean temozolomide concentrations. Plasma temozolomide systemic concentrations unchanged with regadenoson treatment (**a**). Non-contrast enhancing brain interstitium (BI) temozolomide concentration over time failed to increase with regadenoson (**b**). Contrast enhancing BI temozolomide concentration demonstrated a slight increase in temozolomide concentration over time (**c**). Error bars represent standard error of mean values

Additional file 1: Table S1 summarizes the plasma and brain interstitial pharmacokinetic data for each patient, accounting for in vitro fractional recovery [26]. We opted to only assess plasma and brain dialysate samples for temozolomide because the active temozolomide metabolite, MTIC, was associated with poor acid stabilization and recovery [26]. Comparing plasma concentrations of temozolomide alone vs. temozolomide with regadenoson, neither the C_max_ nor AUCs were impacted by regadenoson (Fig. 2, Additional file 2: Fig S1). Peak temozolomide plasma concentrations with temozolomide alone or combined with regadenoson were: 3.5 ± 1.6 µg/ml vs. 4.8 ± 1.2 µg/ml, respectively. Non-contrast enhancing brain concentrations for temozolomide alone or combined with regadenoson were: 0.55 ± 0.26 µg/ml vs. 0.57 ± 0.32 µg/ml, respectively. The non-contrast enhancing brain:plasma AUC ratio was 19.1 ± 10.7% when temozolomide was administered alone and 18.0 ± 7.8% when combined with regadenonson Three patients (patients 2, 3 and 5) demonstrated a rise in non-contrast enhancing mean brain AUC with regadenoson by approximately 53%. But this increase can be mostly attributed to patient 2 who demonstrated a significant rise in temozolomide brain AUC concentration with regadenoson; doubling AUC from 0.6 to 1.2 µg/ml h. Overall, evaluation of non-enhancing brain interstitium mean concentrations for all 5 patients demonstrated no significant difference in C_max_ or AUCs between treatment groups, but individual variations existed.

**Fig. 3:**
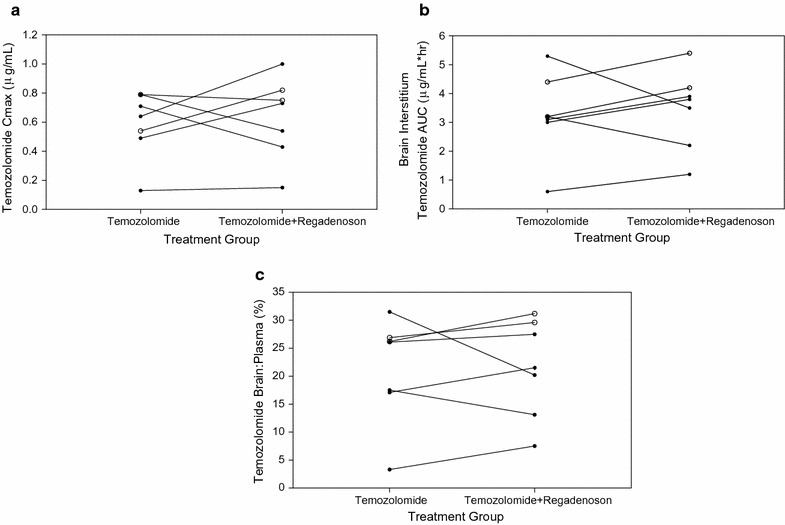
Maximum temozolomide concentration (C_max_) and area under the curve (AUC). C_max_ of contrast enhancing and non-contrast enhancing tissue (**a**). Brain AUC of contrast enhancing and non-contrast enhancing tissue (**b**). Brain:Plasma AUC of contrast enhancing and non-contrast enhancing tissue (**c**). Black circles represent non-contrast enhancing tissue, white circles represent contrast-enhancing tissue

Two patients (patients 1 and 3) had catheters placed in contrast enhancing brain. The dialysate AUCs increased slightly by 10.0 and 19.1% when temozolomide was administered with regadenoson. For patient 1, brain AUC increased from 4.4 to 5.4 µg/ml h with temozolomide alone to combination with regadenoson, respectively. And for patient 3, brain AUC increased from 3.2 to 4.2 µg/ml h with temozolomide alone to combination with regadenoson, respectively. Generally, treatment with regadenoson exhibited a quicker rise to peak concentration but failed to demonstrate a prolonged increase of brain interstitial temozolomide concentrations (Fig. 2). The variations in peak time, T_max_ and AUC can be seen in individual patient neuropharmacokinetics with temozolomide alone vs. temozolomide + regadenoson (Fig. 3, Additional file 1: Table S1).

## Discussion

Despite numerous clinical studies using chemotherapeutic agents, novel biologics and immunotherapeutic agents, the overall survival of patients with high grade gliomas has not changed drastically over the last decade [37]. The clinical impact of many cytotoxic agents has likely been limited in patients with malignant gliomas by their inability to cross the BBB. This poses an issue not only for primary brain tumors but also for metastatic brain disease. Unfortunately, while systemic therapy options have improved over the years for solid tumors, metastatic tumor cells are able to invade the CNS and proliferate with shelter from an impermeable BBB. Thus, with a lack of effective drug entry of varied chemotherapy agents, there has been no improvements in the overall survival of both malignant glioma and metastatic brain tumors. With this small clinical study, we evaluated regadenoson as a tool to facilitate CNS entry of a mildly permeable agent, temozolomide, from the proposed mechanism of enabling transient BBB disruption.

Overall, our study failed to demonstrate that brain interstitial temozolomide concentrations were increased by use of standard dose regadenoson; which we pre-specified as an increase in temozolomide brain concentration by ≥ 50%. Importantly, regadenoson did not alter temozolomide plasma concentrations which could result in changes in temozolomide related efficacy or toxicity. Although these results are consistent with our previous negative imaging study [25], they are at odds with the preclinical data that demonstrated increased drug delivery with one small dose of regadenoson [16, 19, 24, 36]. This difference in effect has raised further questions regarding BBB differences between mice and humans relating to expression and function of CNS adenosine A_2A_ receptors. Alternatively, activation of A_2A_ receptors and subsequent BBB disruption in the brains of glioblastoma patients may differ from the activation of A_2A_ receptors in the normal brain vasculature. Previous preclinical studies demonstrated regadenoson’s ability to decrease cell–cell adhesion integrity while potentially modifying efflux transporter expression within 0.5–2 h after administration [19, 36, 38]. While we anticipated that regadenoson might increase drug entry across the BBB, drug exit from the CNS could also be facilitated resulting in decreased temozolomide concentrations in brain interstitium. The effect on transport is further compounded by the studies by that demonstrated temozolomide’s ability to bind to the multi-drug resistance protein, P-glycoprotein; which likely plays a significant role in glioblastoma temozolomide resistance [39]. Interestingly, regadenoson has been shown to downregulate P-glycoprotein expression in brain endothelial cells thus increasing CNS drug delivery in in vitro *BBB* models and non-tumor bearing rodents [36]. Thus, these combined findings add to the plausibility of temozolomide efflux by P-glycoprotein along with inadequate P-glycoprotein inhibition within brain/brain tumor parenchyma by regadenoson, thereby not causing a significant increase in brain interstitial temozolomide concentrations.

The early rapid rise of temozolomide seen with regadenoson administration can be attributed to the fast acting modulation that results from adenosine receptor activation [16, 24, 36]. Preclinical studies demonstrated the effect of regadenoson on brain vasculature with 0.05 mg/kg dosage per mouse (human equivalent dosing of 0.004 mg/kg); which is less than the standard cardiac stress dosing of 0.006 mg/kg per patient. Yet, despite these preclinical studies utilizing lower than standard regadenoson dosing, increased CNS penetration of 70 kD dextran and temozolomide was observed [16, 19, 24]. Interestingly, these studies in rodents demonstrated a bell shaped dose/effect curve, suggesting that regadenoson doses too high or too low result in minimal changes in BBB disruption. With this clinical study, we opted to use the standard clinical dosing of regadenoson (0.4 mg). This FDA approved agent is used daily in the clinical setting of patients with suspected heart disease to induce vasodilation. Clinically, patients receive one dose with associated cardiac imaging. We hypothesized that because approximately 26% of patients with suspected cardiac disease experience brief headaches after regadenoson administration, it is possible that headaches may be a direct correlate/biomarker for the presence or degree of BBB disruption. We opted to start with the clinical dosing of regadenoson as a means to increase temozolomide CNS entry. While optimal dosing has been determined for cardiac stress testing, optimal dosing and schedule of administration has yet to be determined with a focus on BBB permeability. Thus, it is plausible that increased or decreased standard regadenoson dosing could optimally augment CNS temozolomide entry. These studies of varied regadenoson dosing impacting the BBB permeability have not been performed to date in humans.

## Conclusions

Given the importance of transiently opening the BBB to facilitate drug entry, further research in this area is desperately needed to improve the outcome of patients with CNS malignancies. For both primary and metastatic tumors, treatment options are very limited and/or exhibit poor sustainability for growth inhibition, and invasion. Several agents have been investigated in the past as a means to transiently “open” the BBB, but very few studies or laboratory investigations are being conducted to identify optimal genes, signaling pathways, or receptors so as to design drugs to influence CNS permeability. Regadenoson may be a potential agent, but more studies are needed to define the optimal dose and dosing schedule with the desired effect on CNS vasculature. These questions, along with the proper dosing and schedule of regadenoson, remain to be further studied, in order to explain our negative findings and improve chances of future success in enhancing transient BBB permeability.

## Additional files

**Additional file 1.** Pharmacokinetics of plasma and brain dialysate sampling post temozolomide and regadenoson.

**Additional file 2.** Individual temozolomide concentrations within non-contrast enhancing brain interstitium on a log based scale (A). Contrast-enhancing brain interstitium values in patients 1 and 3 (B). Solid line demonstrates treatment with temozolomide alone. Dashed line demonstrates combined treatment with regadenoson.

## Abbreviations

CNS: central nervous system
BBB: blood brain barrier
MDC: microdialysis catheter
SPECT: single-photon emission computed tomography
AUC: area under the curve
MTIC: methyl-triazenyl imidazole carboxamide
CSF: cerebral spinal fluid
MRM: multiple reaction monitoring
