# Supplementary material for: The effect of an adenosine A2A agonist on intra-tumoral concentrations of temozolomide in patients with recurrent glioblastoma
Source: Fluids Barriers CNS. 2018 Jan 15;15:2. doi: 10.1186/s12987-017-0088-8 (PMC5767971; doi:10.1186/s12987-017-0088-8)
Supplement: Supplementary file 1 — Additional file 1. Pharmacokinetics of plasma and brain dialysate sampling post temozolomide and regadenoson. [file 12987_2017_88_MOESM1_ESM.docx]

Additional Table S1. Pharmacokinetics of plasma and brain dialysate sampling post temozolomide and regadenoson.

| A. Individual patient plasma and non-contrast enhancing brain dialysate pharmacokinetic parameters  **Temozolomide Alone** | | | | | | | | | | |
| --- | --- | --- | --- | --- | --- | --- | --- | --- | --- | --- |
|  | Plasma | | | | Non-contrast enhancing Brain | | | | Brain:Plasma | |
| **Patient** | C_max_ (ug/mL) | T_max_ (hr) | AUC_0-18 hr_ (hr*ug/mL) | t_1/2_  (hr) | C_max_ (ug/mL) | T_max_  (hr) | AUC_0-18 hr_ (hr*ug/mL) | t_1/2_  (hr) | C_max_  (%) | AUC_0-18 hr_  (%) |
| **1** | 2.0 | 3.00 | 16.8 | 2.8 | 0.79 | 7.08 | 5.3 | 2.1 | 39.3 | 31.5 |
| **2** | 5.7 | 1.00 | 18.4 | 1.7 | 0.13 | 9.08 | 0.6 | 7.3 | 2.3 | 3.3 |
| **3** | 2.7 | 3.00 | 11.9 | 1.7 | 0.64 | 3.57 | 3.1 | 1.5 | 23.7 | 26.1 |
| **4** | 4.8 | 2.03 | 18.3 | 1.7 | 0.71 | 3.58 | 3.2 | 1.5 | 14.8 | 17.5 |
| **5** | 2.3 | 8.03 | 17.5 | 2.4 | 0.49 | 7.11 | 3.0 | 1.5 | 21.3 | 17.1 |
| **Median (range)** |  | 3.00  (1.00-8.03) |  |  |  | 7.08  (3.57-9.08) |  |  |  |  |
| **Avg±SD (%CV)** | 3.5±1.6 (47.1%) |  | 16.6±2.7 (16.3%) | 2.1±0.5 (24.9%) | 0.55±0.26  (47.2%) |  | 3.0±1.7 (54.8%) | 2.8±2.5 (91.4%) | 20.3±13.6 (66.7%) | 19.1±10.7 (56.1%) |

| **Temozolomide/Regadenoson** | | | | | | | | | | |
| --- | --- | --- | --- | --- | --- | --- | --- | --- | --- | --- |
|  | Plasma | | | | Non-contrast enhancing Brain | | | | Brain:Plasma | |
| **Patient** | C_max_ (ug/mL) | T_max_ (hr) | AUC_0-18 hr_ (hr*ug/mL) | t_1/2_  (hr) | C_max_ (ug/mL) | T_max_  (hr) | AUC_0-18 hr_ (hr*ug/mL) | t_1/2_  (hr) | C_max_  (%) | AUC_0-18 hr_  (%) |
| **1** | 3.2 | 4.00 | 17.3 | 3.3 | 0.54 | 5.08 | 3.5 | 2.1 | 16.9 | 20.2 |
| **2** | 6.4 | 1.00 | 16.0 | 1.5 | 0.15 | 3.62 | 1.2 | 5.2 | 2.3 | 7.5 |
| **3** | 4.5 | 1.97 | 14.2 | 1.7 | 1.00 | 2.58 | 3.9 | 2.2 | 22.2 | 27.5 |
| **4** | 4.4 | 4.00 | 16.8 | 1.7 | 0.43 | 5.08 | 2.2 | 1.5 | 9.8 | 13.1 |
| **5** | 5.3 | 1.02 | 17.7 | 1.6 | 0.73 | 1.57 | 3.8 | 0.9 | 13.8 | 21.5 |
| **Median (range)** |  | 1.97 (1.00-4.00) |  |  |  | 3.62  (1.57-5.08) |  |  |  |  |
| **Avg±SD (%CV)** | 4.8±1.2 (24.9%) |  | 16.4±1.4 (8.4%) | 2.0±0.8 (38.5%) | 0.57±0.32 (56.0%) |  | 2.9±1.2 (40.3%) | 2.4±1.7 (69.8%) | 13.0±7.5 (57.7%) | 18.0±7.8 (43.3%) |

B. Individual patient plasma and contrast enhancing brain dialysate pharmacokinetic parameters

| **Temozolomide** | | | | | | |
| --- | --- | --- | --- | --- | --- | --- |
|  | Contrast enhancing Brain | | | | Brain:Plasma | |
| **Patient** | Cmax (ug/mL) | Tmax  (hr) | AUC_0-18_ (hr*ug/mL) | t_1/2_  (hr) | Cmax  (%) | AUC_0-18_  (%) |
| **1** | 0.79 | 7.08 | 4.4 | 2.2 | 39.5 | 26.2 |
| **3** | 0.54 | 3.57 | 3.2 | 2.1 | 20.0 | 26.9 |

| **Temozolomide/Regadenoson** | | | | | | |
| --- | --- | --- | --- | --- | --- | --- |
|  | Contrast enhancing Brain | | | | Brain:Plasma | |
| **Patient** | Cmax (ug/mL) | Tmax  (hr) | AUC_0-18_ (hr*ug/mL) | t_1/2_  (hr) | Cmax  (%) | AUC_0-18_  (%) |
| **1** | 0.75 | 5.08 | 5.4 | 3.2 | 23.4 | 31.2 |
| **3** | 0.82 | 2.58 | 4.2 | 2.6 | 18.2 | 29.6 |
