# Supplementary material for: The effect of an adenosine A2A agonist on intra-tumoral concentrations of temozolomide in patients with recurrent glioblastoma
Source: Fluids Barriers CNS. 2018 Jan 15;15:2. doi: 10.1186/s12987-017-0088-8 (PMC5767971; doi:10.1186/s12987-017-0088-8)
Supplement: Supplementary file 2 — Additional file 2. Individual temozolomide concentrations within non-contrast enhancing brain interstitium on a log based scale (A). Contrast-enhancing brain interstitium values in patients 1 and 3 (B). Solid line demonstrates treatment with temozolomide alone. Dashed line demonstrates combined treatment with regadenoson. [file 12987_2017_88_MOESM2_ESM.docx]

Additional Figure S1.

A.

B.
